# Is *Andrographis paniculata* extract and andrographolide anaphylactic?

**DOI:** 10.1016/j.toxrep.2017.07.004

**Published:** 2017-07-29

**Authors:** Edwin Jothie Richard, Sasikumar Murugan, Bharathi Bethapudi, Ramanaiah Illuri, Deepak Mundkinajeddu, Chandrasekaran Chinampudur Velusami

**Affiliations:** Department of Biology, R&D Centre, Natural Remedies Private Limited, Bangalore, 560 100, Karnataka, India

**Keywords:** *Andrographis paniculata* extract, Andrographolide, Active systemic anaphylaxis, Anaphylactoid, &beta, &minus, Hexosaminidase, Leukotriene C_4_

## Abstract

*Andrographis paniculata, “King of bitters”* is a popularly known medicinal plant extensively used in many parts of the world for treatment of various diseases. Since recent past, anaphylactic/allergic type adverse events were reported upon *A. paniculata* usage, the study aimed to evaluate the anaphylactic and anaphylactoid potential of *A. paniculata* extract and andrographolide (a major phytoactive of *A. paniculata*). The anaphylactic potential was evaluated using active systemic anaphylaxis (ASA) assay in guinea pigs. Further, the release of allergic mediators was measured in immunoglobulin E (IgE) sensitized and non-IgE sensitized Rat Basophilic Leukemia (RBL-2H3) cell lines *in-vitro*. *A. paniculata* extract or andrographolide sensitized guinea pigs following the challenge antigen administration orally and intravenously did not demonstrate any clinical signs of anaphylaxis. IgE sensitized and non- IgE sensitized RBL-2H3 cells treated with *A. paniculata* extract did not induce release of allergic mediators. Whereas IgE sensitized and non- IgE sensitized RBL-2H3 cells treated with andrographolide demonstrated mild to moderate release of allergic mediators. *A. paniculata* extract has no anaphylactic and anaphylactoid potential in *in-vivo* and *in-vitro* studies. Whereas, andrographolide effects on allergic mediators in *in-vitro* studies needs to be scrutinized if they are of biologically important.

## Highlights

• *A. paniculata* extract and andrographolide exhibit no signs of anaphylaxis in active systemic anaphylaxis assay.
• *A. paniculata* extract did not release allergic mediators in IgE sensitized and non-sensitized cells.
• Andrographolide demonstrated mild to moderate release of allergic mediators.
• *A. paniculata* has no anaphylactic and anaphylactoid potential in *in-vivo* and *in-vitro* studies.

## Graphical abstract

**Figure:**
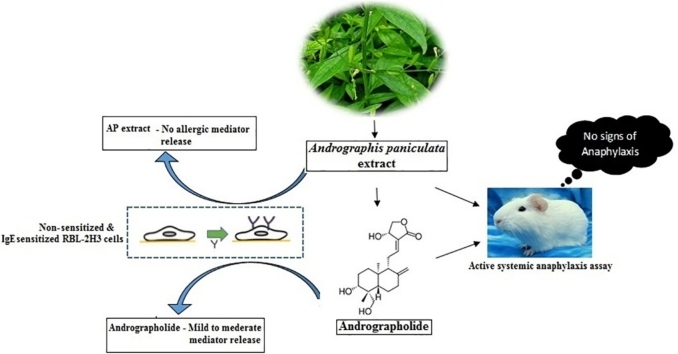

## 1 Introduction

*Andrographis paniculata* (Burm. F.) Nees belonging to family Acanthaceae is the most popular traditionally known medicinal plant used for the treatment of array of disease like viral fever, chicken pox, common cold, diarrhoea, dysentery, eczema, epidemic encephalitis B, hepatitis, herpes zoster, mumps, ulcer, neurodermatitis, inflammation, pharyngolaryngitis, pneumonia, respiratory infections [1], [2]. The plant is widely used as a traditional medicine in countries like India, China, Hongkong, Pakistan, Bangladesh, Malaysia, Philippines, Indonesia and Thailand. It is also used to treat insect, bug and snake bites [1], [3]. It is commonly known as Kalmegh or King of bitters cultivated in many regions of South Asian countries because of well-known medicinal value [4].

In the Ayurvedic system of medicine, *A. paniculata* is often used in combination with other herbs and health care products for treating patients suffering from various physical and mental disorders. It has been estimated that *A. paniculata* is used in more than 50% of herbal compositions commercialized in India for hepatic disorders [5].

*A. paniculata* has been shown to possess wide spectrum of pharmacological properties *viz.*, anti-microbial, anti-cancer, anti-inflammatory, anti-oxidant, immunostimulant, anti-diabetic, anti-infective, hepato-renal protective, anti-angiogenic, anti-allergic etc [4], [6]. The WHO monograph mentions its use for prophylaxis and symptomatic treatment of upper respiratory tract infection, bronchitis, pharyngotonsilitis, urinary tract infections and acute diarrhoea supported by clinical data [7]. *A. paniculata* is rich in labdane diterpinoids. The pharmacological effects of *A. paniculata* have been attributed to the major bitter tasting secondary metabolite *i.e*, andrographolide, a labdane diterpinoid [8].

The published systematic reviews indicate that *A. paniculata* has immense potential for treating various diseases as per traditional and modern systems of medicine [4], [8]. Although, *A. paniculata* is vastly reported to possess safety and efficacy [9], Therapeutic Goods Administration (TGA) has reviewed the pharmacoepidemiological information on the adverse reactions associated with *A. paniculata* usage and reported an association between anaphylactic/allergic-type adverse drug reactions (ADRs) for products that contain *A. paniculata* as an ingredient. However, TGA could not conclude on particular type of extract or quantity of Andrographis to be causative for allergic/antigenic reactions associated with *A. paniculata* products [10]. Hence the present study was performed to evaluate the anaphylactic and anaphylactoid potential of *A. paniculata* extract and its major phytoactive andrographolide using *in-vivo* and *in-vitro* assays.

The present study investigated the anaphylactic potential of *A. paniculata* extract and andrographolide in active systemic anaphylaxis (ASA) assay using guinea pigs. Further, the anaphylactic and anaphylactoid potential was investigated by measuring the release of allergic mediators such as histamine, β-hexosaminidase, leukotriene C_4_ (LTC_4_) and tryptase in IgE sensitized and non-IgE sensitized rat basophilic leukemia (RBL-2H3) cell line.

## 2 Materials and methods

### 2.1 Preparation of *A. paniculata* extract

Coarse ground leaves of *A. paniculata* (300 kg) were extracted with methanol under reflux. Thick paste obtained was dried under vacuum (≤65 °C), milled and sieved (#40) to get a uniform powdered extract of *A. paniculata* (18 kg). To the marc contained in the extractor, water was added and extracted under reflux. The concentrated liquid was then spray dried to get the water extract of *A. paniculata* (10 kg). The alcohol and water extracts were then analyzed for its active constituents and blended to get *A. paniculata* extract. The composition manufactured by Natural Remedies Private Limited known as KalmCold™/Ap-Bio™ adheres to the international quality requirements which include analysis of solvent residue, heavy metals residue, mycotoxin residue, pesticide residue evaluation and microbial contamination.

#### 2.1.1 Analysis

*A. paniculata* extract on HPLC analysis was found to contain the following constituents, *viz.*, andrographolide (>30.0% w/w), isoandrographolide (>0.3% w/w), neoandrographolide (>1.0% w/w), andrograpanin (>0.3% w/w), 14-deoxy-11,12-didehydroandrographolide (≤5.0% w/w), skull-capflavone I (>0.05% w/w) and 7-O-methylwogonin(>0.05% w/w).

### 2.2 Isolation of andrographolide

**Fig. 1:**
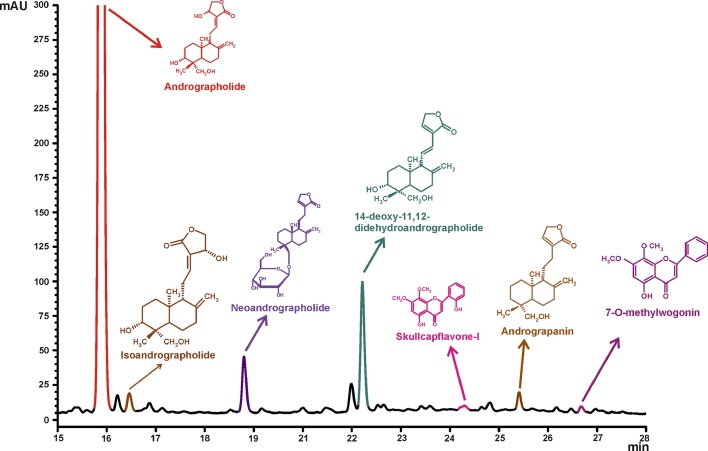
HPLC chromatogram of *A. paniculata* extract.

The extract was subjected to liquid- liquid partitioning between ethyl acetate and water. The ethyl acetate layer was repeatedly chromatographed over silica gel using combinations of hexane: ethyl acetate and chloroform: methanol. Crystallisation of different chromatographic fractions yielded andrographolide. Identification of andrographolide was confirmed by comparing their 1H and 13C NMR data with literature. Purity of isolated compound was determined by HPLC [11] and found to be >98.0%. The HPLC chromatogram is provided in Fig. 1.

### 2.3 *In-vivo* ASA in Guinea pigs

#### 2.3.1 Chemicals

Ovalbumin, Complete Freund’s Adjuvant (CFA) from Sigma Aldrich, USA; dimethyl sulfoxide (DMSO) and sodium chloride from Himedia Laboratories Pvt. Ltd., India were obtained. Mixture of ovalbumin and CFA was used as a positive control which was dissolved in normal saline, while 10% DMSO was used as a vehicle to dissolve the test substances.

#### 2.3.2 Animals

Male Dunkin-Hartley guinea pigs, 6–7 weeks of age were procured from Geniron Biolabs Pvt. Ltd., Bangalore. Guinea pigs were acclimatized for 7 days under optimal temperature of 25 ± 2 °C and 30–70% relative humidity before the initiation of the experimentation. Guinea pigs were allowed free access to feed pellets (VRK Nutritional solutions) and UV purified water *ad libitum*. All the animal procedures were approved by Institutional Animal Ethics Committee of Natural Remedies Pvt. Ltd., Bangalore (IAEC/NR-PCL/02/11.16).

#### 2.3.3 Experimental procedure

The study design including the sensitization and challenge schedule is briefed in Table 1. During sensitization, *A. paniculata* extract (25 mg/kg), andrographolide (8 mg/kg) and DMSO (10 ml/kg) were orally administered to respective groups for 5 days. While for positive control, mixture of ovalbumin and CFA was administered subcutaneously weekly once for 3 weeks [12]. Dose of *A. paniculata* extract, andrographolide for sensitization corresponds to double the clinical dose of *A. paniculata* extract *i.e.*, 200 mg/day.

**Table 1:** Sensitization and challenge schedule of guinea pigs.

Group	Sensitization				ASA Challenge	
	No. of animals	Substance	Dose per day per animal	No. of days	Substance	Dose per day per animal
Test Group	5	*A. paniculata* extract	25 mg/kg (*p.o*)	5	*A. paniculata* extract	12.5 mg/kg (*p.o*)
Test Group	5	*A. paniculata* extract	25 mg/kg (*p.o*)	5	*A. paniculata* extract	2.5 mg/kg (*i.v*)
Test Group	5	Andrographolide	8 mg/kg (*p.o*)	5	Andrographolide	4 mg/kg (*p.o*)
Test Group	5	Andrographolide	8 mg/kg (*p.o*)	5	Andrographolide	0.8 mg/kg (*i.v*)
Vehicle Control	5	DMSO	10 ml/kg (*p.o*)	5	DMSO (10%)	10 ml/kg (*p.o*)
Vehicle Control	5	DMSO	10 ml/kg (*p.o*)	5	DMSO (1%)	1 ml/kg (*i.v*)
Positive Control	5	Ovalbumin + CFA	2.5 mg/kg (*s.c*)	3	Ovalbumin	1.67 mg/kg (*i.v*)

**Sensitization**: *A. paniculata*, Andrographolide, DMSO were administered every day for 5 days continuously; Ovalbumin + CFA administered once in a week for 3 weeks.

**Challenge**: Following sensitization, animals in all the groups will be left untreated and will be challenged with respective challenge antigen as a single dose.

DMSO: Dimethyl sulfoxide; CFA: Complete Freund's Adjuvant; *p.o*:- per oral; *s.c*: sub-cutaneous; *i.v*: intravenous.

Two weeks following the final sensitization, the challenge antigen was either given orally or intravenously into the leg vein. Treatment groups were challenged with *A. paniculata* extract or andrographolide, while the vehicle control and positive control groups were challenged with DMSO and ovalbumin respectively as presented in Table 1.

Clinical sign observations were performed 30 min to 3 h post challenge dose administration. The anaphylactic signs observed were scored according to Lee et al., Kouchi et al., Park et al. [12], [13], [14] as below:

(−): No signs of anaphylaxis/Asymptomatic

(±: Mild): Urination, evacuation, licking or rubbing the nose and ruffling the fur on occasion

(+: Moderate): Cough, sneezing, weakness, restlessness and rales in addition to the above signs

(++: Severe): Piloerection, nostril discharge, lacrimation, salivation, nasal bleeding, convulsion, dyspnoea, staggering gait, rhonchus, cyanosis, side position, flattening, prostration, retching, labored respiration in addition to the above signs

(+++): Death

### 2.4 *In-vitro* studies

#### 2.4.1 Culture conditions

RBL-2H3 rat basophilic leukaemia (CRL-2256™) cell lines were obtained from American Type Culture Collection (ATCC) (Rockville, MD, USA). RBL-2H3 cells was cultured in EMEM conditioned with 15% heat inactivated FBS (HI-FBS) at 37 °C in a humidified incubator (5% CO_2_, 95% air).

#### 2.4.2 Cytotoxicity assay

RBL-2H3 cells were harvested and transferred into 96-well microplates (1 × 10^4^ cells/well). After overnight culturing, cells were cultured with test items for 1 h at 37 °C [15]. Thereafter, the cells were washed and incubated for 1 h with MTT. The optical density was measured using a microplate reader (Molecular Devices, USA) at a wavelength of 570 nm.

#### 2.4.3 Evaluation of sensitized RBL-2H3 cell exposure to *A. paniculata* extract and andrographolide

RBL-2H3 cells were seeded at a density of 1 × 10^5^ cells/well on a 24-well microplate in 0.5 ml of complete medium/well and incubated overnight at 37 °C, 5% CO_2_, 95% air. The cells were sensitized with 0.5 μg/ml of anti-dinitrophenyl (DNP) IgE for 24 h at 37 °C. IgE sensitized cells were treated with solvent control (DMSO 0.1%), or test substance at non-cytotoxic concentrations for 10 min. Cells were incubated with phosphatidylserine (10 μg/ml) for 5 min and followed by 0.5 μg/ml of 2,4-Dinitrophenyl hapten conjugated to bovine serum albumin (DNP-BSA) as antigen for 30 min. Supernatant was collected to quantify histamine, LTC_4_, β – hexosaminidase and tryptase. The quantification of histamine and LTC_4_ levels were carried out by means of homogenous time resolved fluorescence (HTRF) according to the procedure described by the kit manufacturer (CisBio, France). Tryptase and β – hexosaminidase levels were measured using ELISA kits (Cloud Clone, USA).

#### 2.4.4 Evaluation of RBL-2H3 cell exposure to *A. paniculata* extract and andrographolide

RBL-2H3 cells were seeded at a density of 1 × 10^5^ cells/well on a 24 – well microplate in 0.5 ml of complete medium/well and incubated overnight at 37° C, 5% CO_2_, 95% air. The cells were treated with treatment medium containing solvent control (DMSO 0.1%), or test substance at non-cytotoxic concentrations for 60 min. The supernatant was collected to quantify histamine, LTC_4_, tryptase and β – hexosaminidase levels.

### 2.5 Statistical analysis

The results were expressed as scores for ASA assay and mean ± standard deviation for *in-vitro* studies. Statistical analysis was performed using one-way analysis of variance, followed by post hoc Dunnett’s test. P-value <0.05 was considered as statistically significant.

## 3 Result

### 3.1 *In-vivo* ASA in Guinea pigs

In the ovalbumin and CFA sensitized group, subsequent challenge dose resulted in severe signs of anaphylaxis *viz.,* piloerection, flattening or side position, convulsions in 3 of the 5 animals. While, the remaining 2 animals died within 30 min to 3 h post ovalbumin administration. Hence, the positive control clearly demonstrated death as well as severe signs of anaphylaxis. In DMSO (vehicle control) sensitized group that served as vehicle control group, only urination and evacuation of faeces were observed following dosing with challenge antigen (Table 2).

**Table 2:** Active systemic anaphylaxis in guinea pigs.

Group			Severity of anaphylaxis				
Sensitizing antigen	Challenging antigen	No. of Animals	Asymptomatic (−)	Mild (±)	Moderate (+)	Severe (++)	Death (+++)
*A. paniculata* extract (25 mg/kg, *p.o*)	*A. paniculata* extract (12.5 mg/kg, *p.o*)	5	4	1	–	–	–
*A. paniculata* extract (25 mg/kg, *p.o*)	*A. paniculata* extract (2.5 mg/kg, *i.v*)	5	1	4	–	–	–
Andrographolide (8 mg/kg, *p.o*)	Andrographolide (4 mg/kg, *p.o*)	5	2	3	–	–	–
Andrographolide (8 mg/kg, *p.o*)	Andrographolide (0.8 mg/kg, *i.v*)	5	0	5	–	–	–
DMSO (10 ml/kg, *p.o*)	DMSO (10 ml/kg, *p.o*)	5	1	4	–	–	–
DMSO (10 ml/kg, *p.o*)	DMSO (1 ml/kg, *i.v*)	5	0	5	–	–	–
Ovalbumin + CFA (2.5 mg/kg, *s.c*)	Ovalbumin (1.67 mg/kg, *i.v*)	5	–	–	–	3	2

Clinical signs of anaphylaxis scored 30 min following challenge administration.

DMSO: Dimethyl sulfoxide; CFA: Complete Freund's Adjuvant; *p.o*: per oral; *s.c*: sub-cutaneous; *i.v*: intravenous.

*A. paniculata* extract and andrographolide sensitized groups did not demonstrate any signs of anaphylaxis following dosing with challenge antigen. Urination and evacuation of faeces were observed in few animals. These were considered normal and cannot be attributed to anaphylactic reactions as the vehicle control also had similar observations (Table 2).

### 3.2 *In-vitro* Studies

#### 3.2.1 Effect of *A. paniculata* and andrographolide on cell viability

The viability of RBL-2H3 cells upon treatment with *A. paniculata* and andrographolide was studied by using MTT reduction assay. The results indicated that these cells were viable after 1 h treatment with increasing concentration of *A. paniculata* (6.25–50 μg/ml) and andrographolide (0.37–10 μg/ml). Hence the non-cytotoxic concentrations of *A. paniculata* and andrographolide were used in further experiments.

#### 3.2.2 Effect of *A. paniculata* extract and andrographolide on sensitized RBL-2H3 cells

##### 3.2.2.1 Effect of *A. paniculata* extract on histamine, β-hexosaminidase, LTC_4_ and tryptase release in IgE sensitized RBL-2H3 cells

**Fig. 2:**
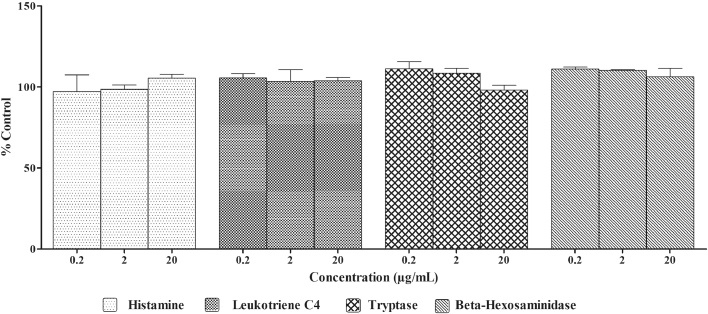
Effect of *A. paniculata* extract on histamine, β-hexosaminidase, LTC_4_ and tryptase release in IgE sensitized RBL-2H3 cells. Anti-DNP IgE sensitized RBL-2H3 cells were treated with different concentrations of *A. paniculata* extract for 10 min. The supernatants from the cells stimulated with DNP-BSA for 30 min were collected, histamine, β-hexosaminidase, LTC_4_ and tryptase released was measured. DMSO was used as blank/solvent control. Histamine, β-hexosaminidase, LTC_4_ and tryptase release rates are shown as mean ± SEM, n = 3.

IgE sensitized RBL-2H3 cells incubated with *A. paniculata* extract at 0.2, 2 and 20 μg/ml dose levels did not induce histamine, β-hexosaminidase, LTC_4_ and tryptase release (Fig. 2).

##### 3.2.2.2 Effect of andrographolide on histamine and LTC_4_ release in IgE sensitized RBL-2H3 cells

**Fig. 3:**
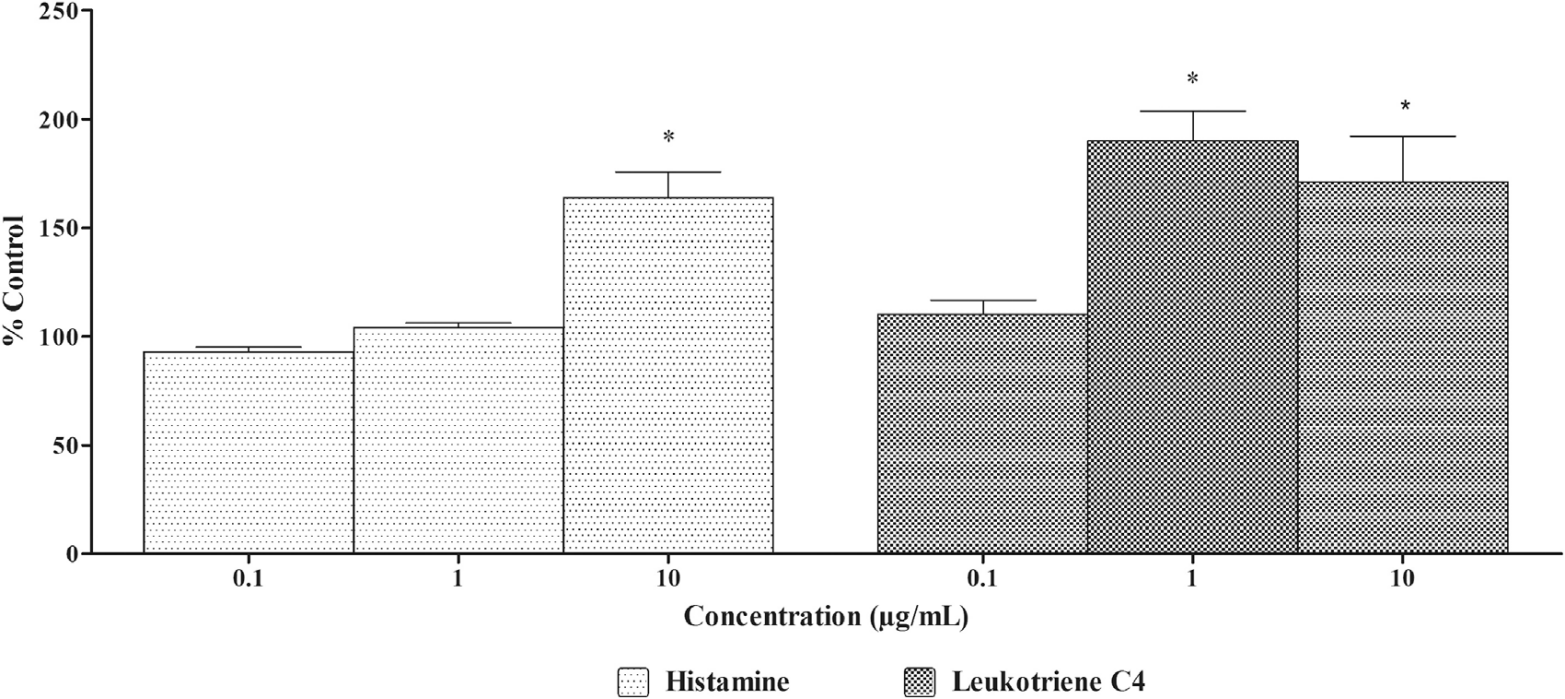
Effect of andrographolide on histamine and LTC_4_ release in IgE sensitized RBL-2H3 cells. Anti-DNP IgE sensitized RBL-2H3 cells were treated with different concentrations of andrographolide for 10 min. The supernatants from the cells stimulated with DNP-BSA for 30 min were collected, histamine and LTC_4_ released was measured. DMSO was used as blank/solvent control. Histamine and LTC_4_ release rates are shown as mean ± SEM, n = 3. An asterisk indicates a significant (*p < 0.05) difference from solvent control.

There was a significant increase in histamine and LTC_4_ levels in IgE sensitized RBL-2H3 cells incubated with andrographolide at higher doses only. Andrographolide treatment did not significantly induce histamine and LTC_4_ release at lower concentrations (Fig. 3).

#### 3.2.3 Effect of *A. paniculata* extract and andrographolide on non- sensitized RBL-2H3 cells

##### 3.2.3.1 Effect of *A. paniculata* extract on histamine and LTC_4_ release in non-IgE sensitized RBL-2H3 cells

**Fig. 4:**
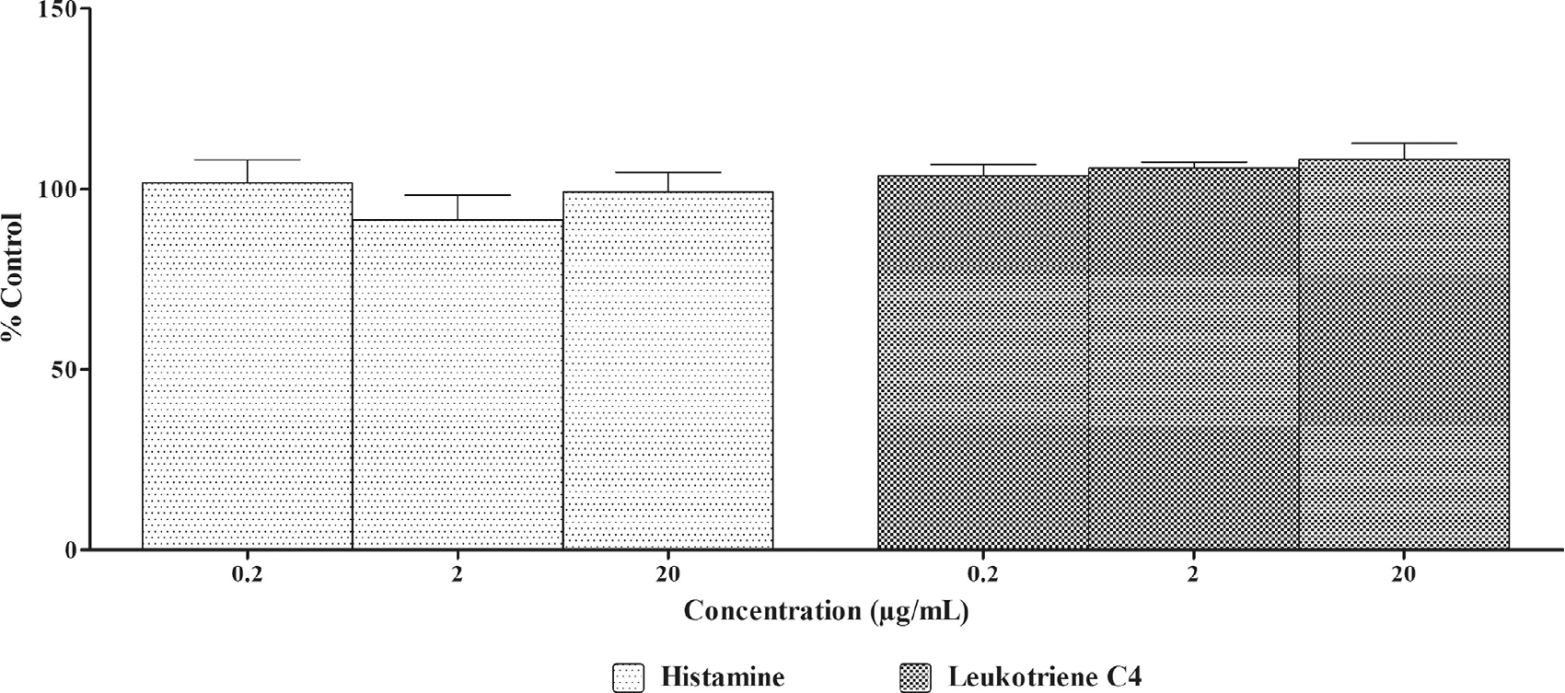
Effect of *A. paniculata* extract on histamine and LTC_4_ release in Non-IgE sensitized RBL-2H3 cells. Non-IgE sensitized RBL-2H3 cells were treated with different concentrations of *A. paniculata* extract for 60 min. The supernatants were collected and histamine and LTC_4_ released was measured. DMSO was used as blank/solvent control. Histamine and LTC_4_ release rates are shown as mean ± SEM, n = 3.

Non-IgE sensitized RBL-2H3 cells treated with *A. paniculata* extract at 0.2, 2 and 20 μg/ml did not induce any significant increase in histamine and LTC_4_ levels when compared with DMSO (Fig. 4).

##### 3.2.3.2 Effect of andrographolide on histamine, β-hexosaminidase, LTC_4_ and tryptase release in non-IgE sensitized RBL-2H3 cells

**Fig. 5:**
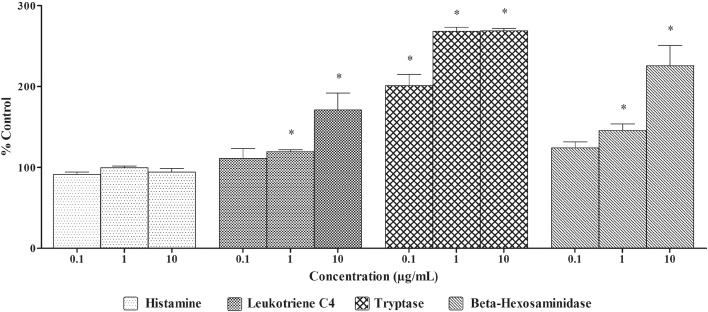
Effect of andrographolide on histamine, β-hexosaminidase, LTC_4_ and tryptase release in Non-IgE sensitized RBL-2H3 cells. Non-IgE sensitized RBL-2H3 cells were treated with different concentrations of andrographolide for 60 min. The supernatants were collected and histamine, β-hexosaminidase, LTC_4_ and tryptase released was measured. DMSO was used as blank/solvent control. Histamine, β-hexosaminidase, LTC_4_ and tryptase release rates are shown as mean ± EM, n = 3. An asterisk indicates a significant (*p < 0.05) difference from solvent control.

Treatment of non-IgE sensitized RBL-2H3 with andrographolide (dose range 0.1–10 μg/ml) did not induce any significant increase in the histamine release when compared to DMSO. While treatment with andrographolide (dose range 0.1–10 μg/ml) except at higher dose (>1 μg/ml) tested, did not induce any significant increase in β-hexosaminidase and LTC_4_ release in comparison to DMSO. However, treatment with andrographolide (dose range 0.1–10 μg/ml) demonstrated significant increase in tryptase levels at all the dose levels tested when compared to DMSO (Fig. 5).

## 4 Discussion

Complementary and alternative medicines (CAM) are gaining popularity globally for various ailments and their usage continues to expand rapidly across the world [16], [17]. *A. paniculata* has immense potential for treating various ailments such as liver disorders, respiratory tract problems and is widely used in many countries. The herb’s efficacy and safety were also profoundly researched [18], [9], [19], [20]. However, hypersensitivity reactions have been reported in the recent past (TGA, 2015). The incidence of allergic/anaphylactic reactions as calculated by an agency Network Nutrition (IMCD), Australia based on their sales data is 0.000024%. However, no definitive evidence has been provided demonstrating the herb as a causative agent of allergic and anaphylactic responses [10]. This has instigated to investigate the anaphylactic and anaphylactoid potential of *A. paniculata* extract as well as for andrographolide.

Anaphylaxis affects one or more organ systems following the exposure to allergen that activates mast cells or basophils via IgE prompting degranulation and immediate release (5–30 min) of preformed mediators such as histamine, leukotrienes, tryptase etc. which are responsible for the occurrence of clinical signs and symptoms of anaphylaxis [21]. One of the assays that is extensively used to study the anaphylactic responses is ASA assay [22]. The current study employed ASA assay to evaluate the anaphylactic potential of *A. paniculata* extract and andrographolide in guinea pigs.

In the ASA assay, positive control sensitized with mixture of ovalbumin and Complete Freund’s Adjuvant followed by ovalbumin challenge demonstrated severe clinical signs of anaphylaxis including death in two animals indicating the reliability of this model for evaluating antigenicity of test substance [12]. The animals orally sensitized with *A. paniculata* extract or andrographolide and subsequently challenged with *A. paniculata* extract or andrographolide orally/intravenously to respective groups did not demonstrate any signs of anaphylaxis. Although urination and defecation were observed in the treatment groups, as they were reported in the vehicle control group and were not accompanied by other moderate or severe anaphylactic signs, these signs do not indicate antigenicity. Thus, the study findings demonstrated no anaphylactic potential of *A. paniculata* extract and andrographolide in ASA assay.

RBL-2H3 cells are used to study comprehensive events on mast cells induced by allergens. The present study employed Anti-DNP IgE sensitized and DNP-BSA stimulated RBL-2H3 cells to investigate the allergic effects of *A. paniculata* extract and andrographolide [23], [24].

The results of treatment of IgE sensitized RBL-2H3 cells with *A. paniculata* extract/andrographolide were compared to DMSO treatment. There was no significant difference between the DMSO and *A. paniculata* extract treated groups. *A. paniculata* extract did not induce release of histamine, β-hexosaminidase, LTC_4_ and tryptase from IgE sensitized RBL-2H3 cells indicating that extract does not have any antigenic potential. However, andrographolide did not induce release of histamine and LTC_4_ from IgE sensitized RBL-2H3 cells at low doses. At higher dose tested andrographolide induced histamine and LTC_4_ release in IgE sensitized RBL-2H3 cells.

Anaphylactoid reactions are non-IgE mediated release of histamine, tryptase and other allergic mediators from mast cells and basophils via different trigger mechanisms [25]. Measurement of histamine and other allergic mediators in non-IgE sensitized RBL-2H3 cells gives an indication if the test substance is having anaphylactoid potential. Treatment with *A. paniculata* extract did not result in degranulation and release of allergic mediators from non-IgE sensitized RBL-2H3 cells. However, andrographolide treatment resulted in release of tryptase and β-hexosaminidase and LTC_4_ at higher doses tested through a non-IgE mediated pathway indicating it might induce anaphylactoid reactions. Hu et al. reported similar anaphylactoid potential for andrographolide in P815 mast cell degranulation model *in vitro* [26]. Although the effects of andrographolide on the allergic mediators are statistically significant in *in-vitro* studies, these effects were not observed in *in-vivo* studies hence, the biological importance of the effects needs to be scrutinized [27]. Hence, a conclusive remark on the anaphylactic and anaphylactoid potential of andrographolide cannot be made.

The study findings indicate that *A. paniculata* extract that is standardized to various constituents has not demonstrated any anaphylactic potential. As reported by Suwankesawong et al. there is a possibility that hypersensitivity reaction might be related to product contamination and its lack of standardization across brands [28]. Hence this factor needs to be considered while making conclusive remarks on the potential relationship between allergic/anaphylactic reactions and *A. paniculata*.

## 5 Conclusion

In conclusion, treatment with *A. paniculata* extract did not induce clinical signs of anaphylaxis in guinea pigs and also did not induce allergic mediators release from IgE sensitized and non-IgE sensitized RBL-2H3 cells indicating that *A. paniculata* extract does not have anaphylactic and anaphylactoid potential under the conditions tested in the present study.

While andrographolide although did not induce clinical signs of anaphylaxis in guinea pigs, it induced allergic mediators release from IgE sensitized and non-IgE sensitized RBL-2H3 cells at higher concentrations however conclusive remark can be made on the anaphylactic potential of andrographolide after scrutinizing biological significance.

## Authors contribution

Study conception and design: Edwin, Bharathi, Deepak.

Acquisition of data: Sasikumar, Edwin, Ramanaiah.

Analysis and interpretation of data: Edwin, Sasikumar, Bharathi, Chandrasekaran.

Drafting of manuscript: Bharathi, Edwin, Ramanaiah, Sasikumar.

Critical revision: Bharathi, Chandrasekaran, Deepak.

## Conflict of interest

The author(s) confirm that this article content has no conflict of interest.
